# Comparative short-term and survival outcomes of three specimen extraction techniques in laparoscopic low rectal cancer surgery: does it affect ileostomy closure?

**DOI:** 10.1186/s12893-023-01995-8

**Published:** 2023-05-11

**Authors:** Haipeng Chen, Fei Huang, Ming Yang, Zhixun Zhao, Xu Guan, Zheng Liu, Zheng Jiang, Qian Liu, Zhaoxu Zheng, Xishan Wang

**Affiliations:** 1 Department of Colorectal Surgery, National Clinical Research Center for Cancer/ Cancer Hospital, National Cancer Center, Chinese Academy of Medical Sciences and Peking Union Medical College, Beijing, China; 2 Department of Colorectal Surgery, National Clinical Research Center for Cancer/Cancer Hospital, National Cancer Center, Chinese Academy of Medical Science and Peking Union Medical College, No. 17 Panjiayuan Nanli, Chaoyang District, Beijing, 100021 China

**Keywords:** Low rectal cancer, Ileostomy closure, Specimen extraction techniques, Laparoscopy

## Abstract

**Introduction:**

This study aimed to compare the short-term and survival outcomes in laparoscopic low rectal cancer surgery with three different specimen extraction techniques, and whether it affects loop ileostomy closure.

**Materials and methods:**

A consecutive series of patients with low rectal cancer who underwent laparoscopic low anterior resection plus protective loop ileostomy (LAR-PLI) were enrolled. Three main techniques, namely specimen extraction through auxiliary incision (EXAI), specimen extraction through stoma incision (EXSI), and specimen eversion and extra-abdominal resection (EVER), were employed. The postoperative short-term and survival outcomes of the three techniques and the impact on loop ileostomy closure were compared.

**Results:**

In all, 254 patients were enrolled in this study: 104 (40.9%) in the EXAI group, 104 (40.9%) in the EXSI group, and 46 (18.1%) in the EVER group. For primary surgery, EXAI group had significantly longer operative time (P < 0.001), more intraoperative bleeding (P < 0.001), longer length of abdominal incision (P<0.001), longer time to first flatus (P < 0.001), longer time to first defecation (P < 0.001), longer time to first eat (P < 0.001), and longer postoperative hospital stays (P = 0.005) than the EXSI and EVER groups. The primary postoperative complication rate in the EXAI and EVER group was significantly higher than in the EXSI group (P = 0.005). In loop ileostomy closure, EXAI group had significantly longer operative time (P = 0.001), more bleeding volume, and longer postoperative hospital stays (P < 0.001) than the EXSI and EVER groups. For survival outcomes, the 3-year local recurrence-free survival (LRFS) is 92.6% for all patients. The 3-year LRFS for patients in EXAI, EXSI, and EVER were 90.1%, 95.4%, and 92.7%, with P = 0.476.

**Conclusions:**

Our single-center results found that in LAR-PLI surgery for low rectal cancer, the short-term outcomes of specimen extraction through the stoma incision or anus were better than that through the auxiliary incision, but the 3-year LRFS was no statistically different.

## Introduction

The latest data from the Chinese National Cancer Center (CNCC) showed that there were 408,000 new cases of colorectal cancer and 196,000 deaths in 2016, colorectal cancer ranks second and fourth, respectively, in terms of morbidity and mortality, with both showing a continuing upward trend [1]. The ratio of colon cancer and rectal cancer in China is close to 1:1, but the proportion of low rectal cancer is high, accounting for about 60–75% of all rectal cancers [2]. Although total mesorectal excision (TME) is the current standard of surgical practice for radical resection of mid-low rectal cancer, the use of protective loop ileostomy remains controversial [3].

With the increasing inclination of patients to preserve the anus and the continuous advancement of surgical techniques such as the clinical application of double stapler technology, low intestinal anastomosis has been greatly improved. Low anterior resection (LAR) with TME has become the most widely used surgery for low rectal cancer. In our center, patients with low rectal cancer routinely undergo LAR plus protective loop ileostomy (LAR-PLI). Objectively, PLI can reduce the complications caused by postoperative rectal anastomotic leakage [4], but the complications caused by the stoma itself cannot be ignored [5], and even 6–32% of temporary stomas cannot be closed [6–10].

Over the past decade, surgical techniques and instrumentation platforms have continued to improve and innovate, and minimally invasive concepts have found widespread application in colorectal surgery [11]. For LAR-PLI, there are various specimen extraction techniques to reduce abdominal trauma. In current clinical practice, the techniques of specimen extraction in LAR-PLI are mainly classified into three types: specimen extraction through auxiliary incision (EXAI), specimen extraction through stoma incision (EXSI), and specimen eversion and extra-abdominal resection (EVER).

However, there is still a lack of relevant research on whether these specimen extraction techniques affect loop ileostomy closure. In our retrospective study, we enrolled patients who underwent LAR-PLI to compare the short-term and survival outcomes of three different specimen extraction techniques, and evaluate whether different specimen extraction methods affected loop ileostomy closure.

## Materials and methods

### Study population

A consecutive series of patients with low rectal cancer who underwent laparoscopic LAR-PLI at the Cancer Hospital of the Chinese Academy of Medical Sciences between April 2015 and September 2019 were enrolled in this retrospective study. The inclusion criteria were: (1) All patients were pathologically diagnosed with rectal adenocarcinoma; (2) The primary tumor was a low rectal cancer (the distance of the tumor from the anal verge measured by colonoscopy ≤ 7 cm); and (3) All patients underwent laparoscopic LAR-PLI and successful loop ileostomy closure. Patients with distant metastasis and recurrent disease; those who underwent transanal local excision or open rectal surgery; those with ASA classification > grade 3; those with a history of other malignancies; and those lost to follow-up were excluded. According to the procedures of specimen extraction, patients were divided into three groups: EVAI group, EXSI group, and EVER group. To assess the safety of LAR-PLI, we analyzed perioperative data including postoperative complications, stoma-related complications, operative time, estimated blood loss, time to first flatus, time to first defecation, time to first eat, time to remove the urinary catheter, length of postoperative hospital stay, 30-day re-operation rate, 30-day mortality, and interval to loop ileostomy closure. The American Joint Committee on Cancer TNM staging standard of rectal cancer (7th edition) was used for staging. The decision regarding preoperative or postoperative adjuvant therapy (capecitabine or CAPOX with or without pelvic radiotherapy) was made according to experienced senior oncologists in our hospital. If the patient died during the follow-up period, it was considered as a censored case.

### Surgical procedures of primary operation

All procedures were conducted by an experienced surgical team, and a modified lithotomy position and five-trocar laparoscopic approach were used. All patients received standard bowel preparation, and prophylactic antibiotics were started 30–60 min before commencement of surgery. The ileostomy site was determined preoperatively by a professional stoma nurse.

The whole surgical procedure [12] was compiled based on TME. It was started at the level of the sacral promontory; the sigmoid colon was dissociated using the medial-to-lateral approach. The origin of the inferior mesenteric artery was ligated at the point of origin, and the regional lymph nodes at the root of the inferior mesenteric artery vessels were dissected. The right and the left lateral attachments of the sigmoid colon were incised cephalically towards the distal part of the descending colon and caudally towards the colorectal junction. For specimen extraction and digestive tract reconstruction, different surgical procedures were followed, according to the type of technique chosen:

**Fig. 1:**
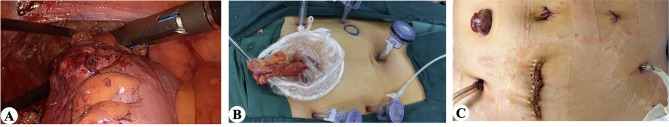
EXAI surgical procedure. (A) The proximal rectal wall is cut off. (B) Extraction of rectal specimen through auxiliary incision. (C) Postoperative abdominal stoma

**Fig. 2:**
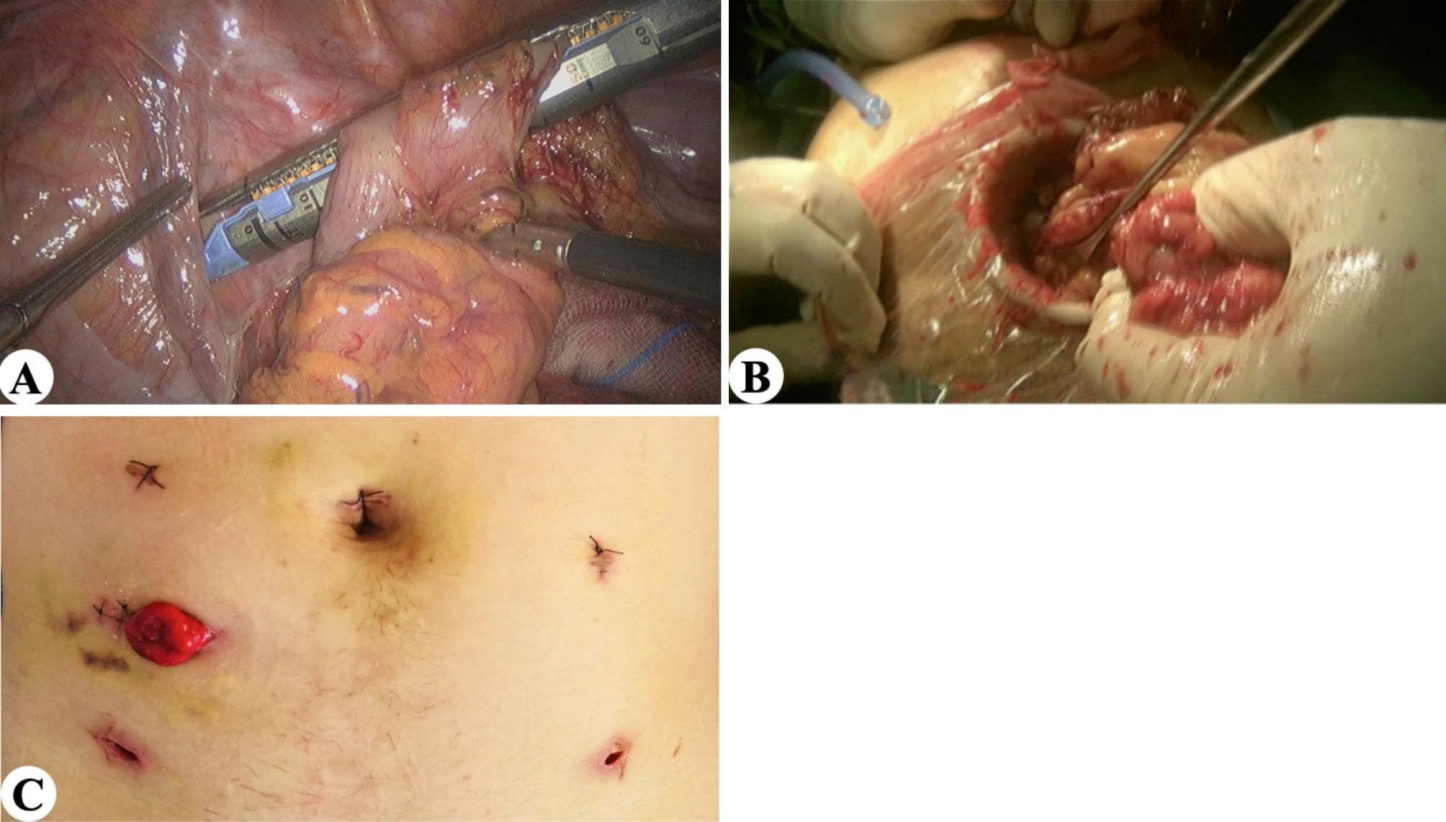
EXSI surgical procedure. (A) The proximal rectal wall is cut off. (B) Extraction of rectal specimen through stoma incision. (C) Postoperative abdominal stoma

EXSI: The proximal bowel can be resected either intra-abdominally or extra-abdominally (Fig. 2A). A 3–5 cm incision is made at the pre-marked stoma site, a sterile plastic cannula is then introduced into the pelvis through the stoma, and the rectal specimen is removed(Fig. 2B). The anvil is introduced into the bowel lumen and closed with a purse string, and an end-to-end anastomosis is performed. Postoperative abdominal wall has trocar holes and stoma incision (Fig. 2C).

**Fig. 3:**
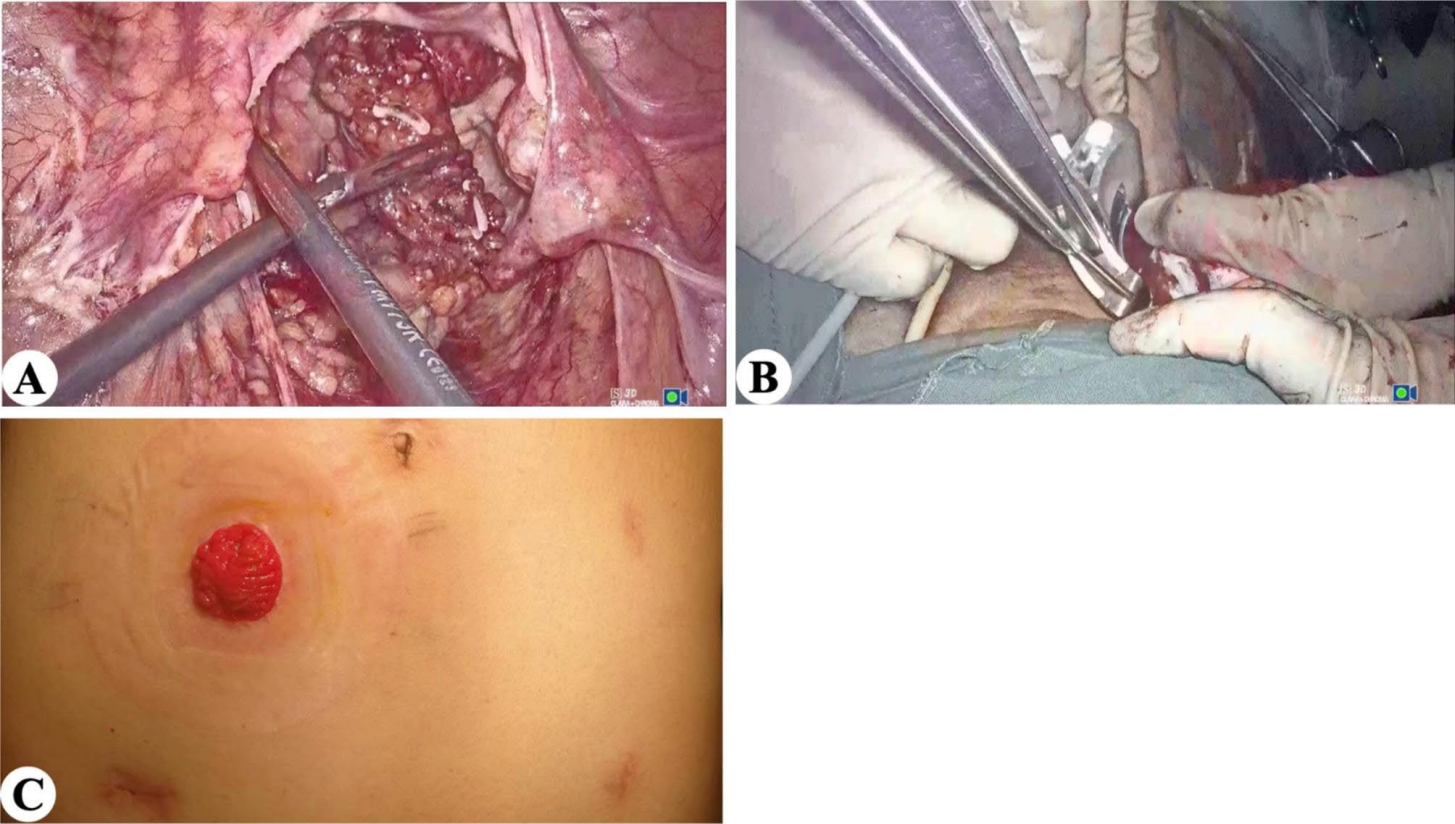
EVER surgical procedure. (A) Rectal specimen is everted extracorporeally. (B) The distal rectal resection is performed. (C) Postoperative abdominal stoma (longer stoma incision)

EVER: After resection of the proximal rectum, use a ring clamp to grasp the rectal stump and gently drag it outside the body (Fig. 3A). the distal rectum is excised using a curved cutter stapler (Fig. 3B). The rectal stump is redelivered into the pelvic cavity, followed by an end-to-end anastomosis. The postoperative abdominal wall only has trocar holes and a stoma (no extended incision) (Fig. 3C).

After reconstruction of the digestive tract, the terminal ileum approximately 30–40 cm proximal to the ileocecal junction was chosen as the stoma position. An appropriate position of the stoma was usually selected in the lower right abdominal wall within the rectus abdominis muscle. The position was determined at the discretion of the chief surgeon according to the patient’s opinions, preoperative stoma marking, intraoperative abdominal wall thickness, and ileal diameter.

### Stoma closure

**Fig. 4:**
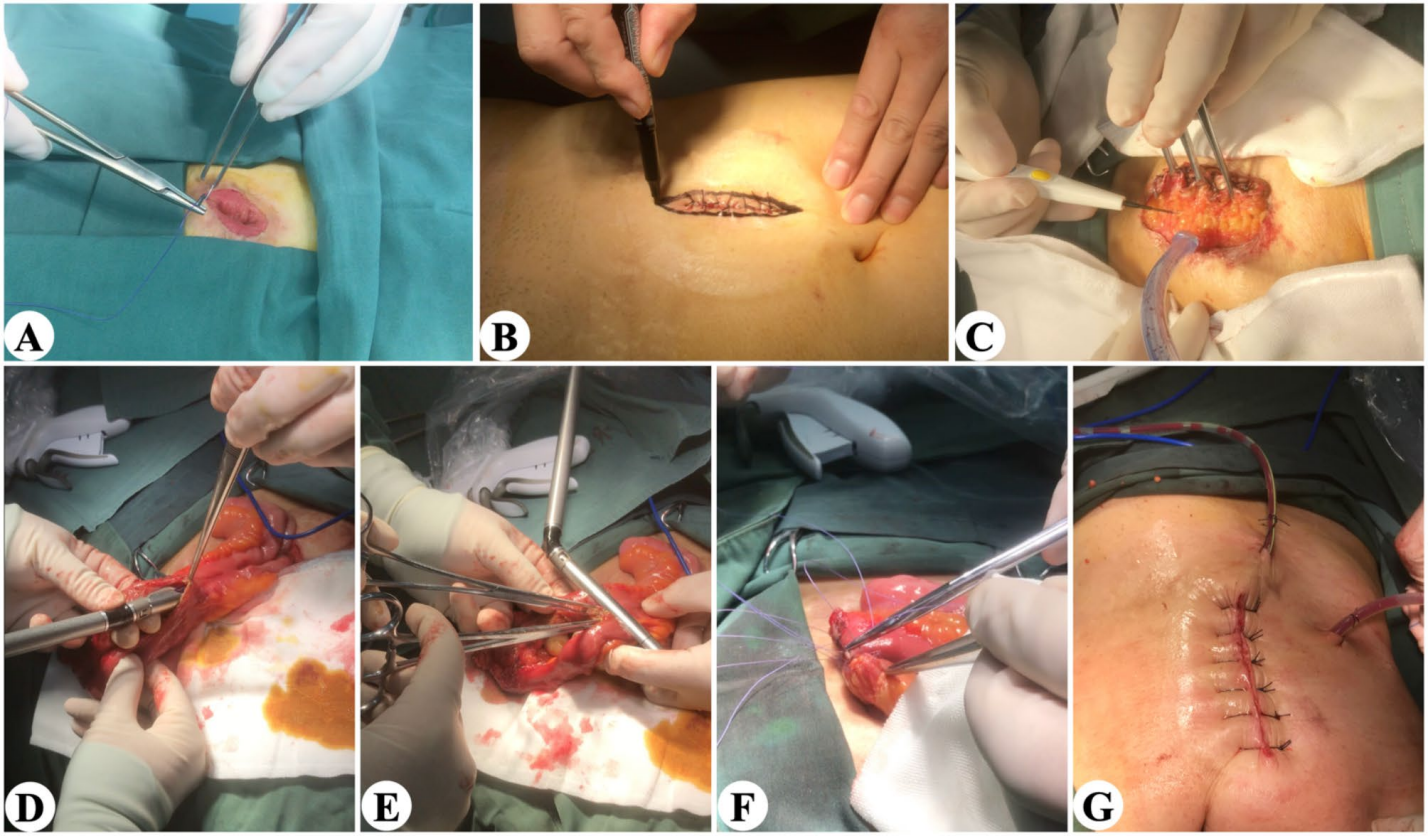
Stoma closure procedure. (A) Suture stoma. (B) Mark the incision. (C) Enter the abdominal cavity layer by layer. (D) Side-to-side anastomosis. (E) Cut the stump. (F) Reinforced anastomosis. (G) Place the drainage tube

Details of stoma-related complications were also recorded. Loop ileostomy closure was usually performed approximately 3–6 months after primary surgery in the absence of complications or adjuvant chemoradiotherapy. For those patients who receive adjuvant chemotherapy or radiotherapy after primary surgery, the ileostomy closure usually occurs about 4 weeks after treatment ends. If the patient has postoperative complications, such as anal dysfunction, the time for ileostomy closure surgery will be longer. The operation was undertaken by the surgeon who performed the primary operation, and the stoma closure was performed by functional end-to-end anastomosis using linear staplers (Fig. 4). Short-term outcomes after stoma closure in each group were analyzed.

### Statistical analysis

The data are presented as a number for categorical variables and the mean ± standard deviation for continuous variables. The chi-square test and Fisher’s exact test were used to evaluate differences in categorical variables, and Student’s *t*-test and analysis of variance (ANOVA) were used to evaluate continuous variables. Two-sided P values < 0.05 were considered to indicate statistical significance. All data analysis was performed using SPSS software (version 26.0, IBM Corporation, Armonk, NY, USA).

## Results

### Patient characteristics

A total of 254 patients were enrolled in this study, including 104 (40.9%) in the EXAI group, 104 (40.9%) in EXSI group, and 46 (18.1%) in the EVER group. Except for the variable distance of the tumor from the anal verge (P = 0.001), there were no statistically significant differences in clinical characteristics between the groups. The distance of the tumor from the anal verge in the EVER group was closer than that in the other groups (P < 0.001), but the mean distance was also greater than the 2-cm safe margin standard. The details are shown in Table 1.

**Table 1:** Comparison of characteristics of all included patients

*Variables*	*EXAI n = 104 n(%)*	*EXSI n = 104 n(%)*	*EVER n = 46 n(%)*	*P value*
Sex				0.875
Male	70 (67.3)	68 (65.4)	29 (63.0)	
Female	34 (32.7)	36 (34.6)	17 (37.0)	
Age				0.700
< 60 y	58 (55.8)	64 (61.5)	27 (58.7)	
≥ 60 y	46 (44.2)	36 (38.5)	19 (41.3)	
BMI, kg/m^2^				0.084
< 25	48 (46.2)	64 (61.5)	25 (54.3)	
≥ 25	56 (53.8)	40 (38.5)	21 (45.7)	
ASA score				0.454
I	6 (5.8)	10 (9.6)	2 (4.3)	
II	95 (91.3)	92 (88.5)	41 (89.1)	
III	3 (2.9)	2 (1.9)	3 (6.5)	
Combined diabetes				0.584
None	70 (67.3)	68 (65.4)	34 (73.9)	
Yes	34 (32.7)	36 (34.6)	12 (26.1)	
Abdominal surgery history				0.917
None	83 (79.8)	81 (77.9)	37 (80.4)	
Yes	21 (20.2)	23 (22.1)	9 (19.6)	
Intestinal obstruction^*^				0.236
None	91 (87.5)	90 (86.5)	44 (95.7)	
Yes	13 (12.5)	14 (13.5)	2 (4.3)	
Neoadjuvant therapy				0.070
None	76 (73.1)	76 (73.1)	41 (89.1)	
Yes	28 (26.9)	28 (26.9)	5 (10.9)	
AJCC pTNM stage				0.159
0	5 (4.8)	4 (3.8)	3 (6.5)	
I	39 (37.5)	25 (24.0)	19 (41.3)	
II	24 (23.1)	38 (36.5)	11 (23.9)	
III	36 (34.6)	37 (35.6)	13 (28.3)	
Tumor size, cm, mean ± SD	3.5 ± 1.7	3.8 ± 1.5	3.2 ± 1.5	0.112
Distance of the tumor from the anal verge, cm, mean ± SD	5.2 ± 1.2	5.0 ± 1.2	4.4 ± 1.1	0.001

EXAI, specimen extraction through the auxiliary incision; EXSI, specimen extraction through the stoma incision; EVER, specimen eversion and extra-abdominal resection; AJCC, American Joint Committee on Cancer; ^*^primary surgery

### Perioperative short-term results

For primary surgery, the EXAI group had significantly longer operative time (P < 0.001), more intraoperative bleeding (P < 0.001), longer length of abdominal incision (P < 0.001), longer time to first flatus (P < 0.001), longer time to first defecation (P < 0.001), longer time to first eat (P < 0.001), and longer postoperative hospital stays (P = 0.005) than EXSI and EVER group (Table 2). The time to remove urinary catheter was significantly shorter in the EXSI group (P < 0.001), and EVER group had the shortest interval to loop ileostomy closure (P = 0.018).

**Table 2:** Perioperative outcomes of surgery

*Variables*		*EXAI* *n = 104*	*EXSI* *n = 104*	*EVER* *n = 46*	*Overall comparison*		*Multiple comparisons*	
						*EXAI vs. EXSI*	*EXSI vs. EVER*	*EXAI vs. EVER*
	*Mean/SD*	*Mean/SD*	*Mean/SD*	*F*	*P*	*P*	*P*	*P*
Primary surgery								
Operative time, min	217.6 ± 53.7	184.0 ± 53.9	197.0 ± 54.0	10.2	< 0.001	< 0.001	0.173	0.032
Bleeding volume, ml	89.4 ± 96.6	52.4 ± 53.5	39.8 ± 27.0	10.5	< 0.001	< 0.001	0.321	< 0.001
Length of abdominal incision, cm	7.4 ± 1.6	1.5 ± 0.8	1.2 ± 0	851.9	< 0.001	< 0.001	0.150	< 0.001
Time to first flatus, d	3.1 ± 1.0	2.5 ± 0.9	2.4 ± 0.7	11.3	< 0.001	< 0.001	0.522	< 0.001
Time to first defecation, d	3.7 ± 1.1	3.2 ± 1.0	2.9 ± 0.9	10.2	< 0.001	0.002	0.095	< 0.001
Time to first eat, d	3.6 ± 1.8	2.8 ± 1.9	2.3 ± 0.9	10.3	< 0.001	0.001	0.124	< 0.001
Time to remove urinary catheter, d	6.0 ± 1.1	4.0 ± 1.3	5.4 ± 0.9	77.7	< 0.001	< 0.001	< 0.001	0.003
Postoperative hospital stays, d	8.6 ± 2.5	7.6 ± 2.7	7.4 ± 1.8	5.4	0.005	0.005	0.729	0.010
Interval to loop ileostomy closure, d	274.7 ± 137.6	243.8 ± 126.4	213.2 ± 91.5	4.1	0.018	0.078	0.170	0.006
Loop ileostomy closure								
Operative time, min	97.7 ± 30.3	82.5 ± 24.7	83.6 ± 39.4	7.8	0.001	< 0.001	0.830	0.009
Bleeding volume, ml	29.5 ± 18.0	21.2 ± 16.5	22.8 ± 22.8	5.7	0.004	0.001	0.607	0.042
Length of stoma incision, cm	8.0 ± 1.6	6.6 ± 1.5	7.4 ± 2.0	17.8	< 0.001	< 0.001	0.011	0.037
Time to first flatus, d	3.0 ± 1.4	3.0 ± 0.8	3.0 ± 1.1	0.1	0.924	0.723	0.981	0.762
Time to first defecation, d	3.6 ± 1.5	3.5 ± 0.8	3.7 ± 1.2	0.3	0.759	0.817	0.460	0.577
Time to first eat, d	2.9 ± 1.6	2.7 ± 0.7	2.7 ± 1.1	1.3	0.279	0.112	0.620	0.452
Time to remove urinary catheter, d	3.9 ± 1.1	3.7 ± 1.0	4.2 ± 1.1	3.6	0.028	0.206	0.008	0.093
Postoperative hospital stays, d	7.0 ± 2.5	5.8 ± 1.7	6.1 ± 2.2	8.7	< 0.001	< 0.001	0.396	0.020

EXAI, specimen extraction through the auxiliary incision; EXSI, specimen extraction through the stoma incision; EVER, specimen eversion and extra-abdominal resection

For loop ileostomy closure, the operative time (P = 0.001), length of stoma incision (P < 0.001), and postoperative hospital stays (P < 0.001) in the EXAI group were significantly longer than EXSI and EVER group, bleeding volume was also higher in the EXAI group (P = 0.004). The time to remove the urinary catheter in the EXSI group was significantly shorter than the other two groups (P = 0.008). Multiple comparisons showed that there was no significant difference in the time to first flatus, first defecation and first eat between the groups (Table 2).

### Complications of primary surgery

The total postoperative complication rate in the EXSI group was significantly lower than that EXAI and EVER group (10.6% vs. 27.9% vs. 26.1%, P = 0.005). The rates of serious complications (Clavien-Dindo ≥ grades 3) in the EXSI/EXAI/EVER groups were 5.8%, 14.4%, and 13.0%, respectively (P = 0.108). In each group, the most common complication was anastomotic stenosis. There was no statistical difference in blood transfusion among the three groups. No 30-day readmission occurred in each group. Stoma-related complications, there was no difference in the total complications among the three groups (32.7% vs. 29.8% vs. 26.1%, P = 0.712). The most common complication was stomal fecal dermatitis (Table 3).

**Table 3:** Postoperative complications related to primary surgery

*Variables*	*EXAI n = 104*	*EXSI n = 104*	*EVER n = 46*	*P*
Total complications (within 30 days after surgery), n (%)	29 (27.9)	11 (10.6)	12 (26.1)	0.005
Anastomotic leakage, n (%)	0 (0)	2 (1.9)	0 (0)	
Anastomotic stenosis, n (%)	13 (12.5)	4 (3.8)	8 (17.4)	
Anastomotic granuloma, n (%)	0 (0)	1 (1.0)	0 (0)	
Anastomotic bleeding, n (%)	1 (1.0)	0 (0)	1 (2.2)	
Abnormal liver function, n (%)	0 (0)	1 (0.9)	0 (0)	
Intestinal obstruction, n (%)	7 (6.7)	2 (1.9)	1 (2.2)	
Parastomal hernia, n (%)	3 (2.9)	1 (1.0)	0 (0)	
Stoma-related bleeding, n (%)	0 (0)	0 (0)	1 (2.2)	
Intraperitoneal infection, n (%)	2 (1.9)	0 (0)	2 (4.3)	
Auxiliary incision complications, n (%)	7 (6.7)	0 (0)	0 (0)	
Chyle leak, n (%)	1 (1.0)	0 (0)	0 (0)	
Postoperative blood transfusion, n (%)	5 (4.8)	1 (1.0)	0 (0)	0.189
30-day readmission, n (%)	0 (0)	0 (0)	0 (0)	
Stoma-related complications (before loop ileostomy closure), n (%)	34 (32.7)	31 (29.8)	12 (26.1)	0.712
Hernia, n (%)	7 (6.7)	5 (4.8)	0 (0)	
Prolapse, n (%)	1 (1.0)	0 (0)	0 (0)	
Retraction, n (%)	3 (2.9)	1 (1.0)	3 (2.9)	
Stenosis, n (%)	1 (1.0)	0 (0)	0 (0)	
Necrosis, n (%)	0 (0)	0 (0)	0 (0)	
Bleeding, n (%)	2 (1.9)	1 (1.0)	1 (1.0)	
Granuloma, n (%)	2 (1.9)	0 (0)	1 (1.0)	
Fecal dermatitis, n (%)	16 (15.4)	21 (20.2)	7 (6.7)	
Skin ulcers, n (%)	1 (1.0)	5 (4.8)	2 (1.9)	
Mucocutaneous detachment, n (%)	2 (1.9)	0 (0)	0 (0)	

EXAI, specimen extraction through the auxiliary incision; EXSI, specimen extraction through the stoma incision; EVER, specimen eversion and extra-abdominal resection; n, number

### Survival outcomes

**Fig. 5:**
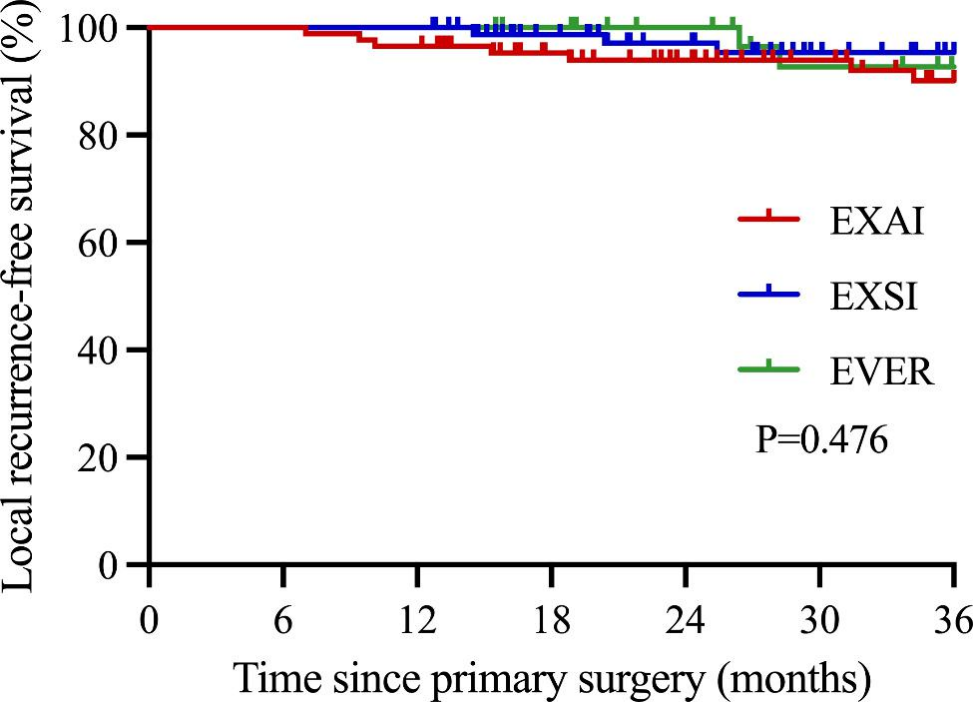
Comparison of 3-year local recurrence-free survival in different techniques

The median local recurrence-free survival (LRFS) time for all patients in this study was 36.0 months. The 3-year LRFS was 92.6% for all patients. The 3-year LRFS for patients in EXAI, EXSI, and EVER were 90.1%, 95.4%, and 92.7%, with P = 0.476 (Fig. 5). The 3-year local recurrence rate (LRR) was 4.7% for all patients. The LRR was 6.7% for patients in EXAI, 2.9% in EXSI, and 4.1% in EVER, with P = 0.491. There was no statistical difference between the groups.

## Discussion

Technological advances and innovations in colorectal surgery over the past few decades have not only reduced abdominal surgical incisions but also avoided specimen extraction sites [13]. However, there are still only few relevant studies on whether different specimen extraction methods affect the short-term curative effect and stoma recovery of patients.

In this study, we only enrolled patients with low rectal cancer who underwent LAR-PLI to evaluate the efficacy and safety of three different specimen extraction procedures by analyzing the short-term postoperative outcomes and stoma-related complications. In terms of surgical procedures, the EXSI and EVER groups had less abdominal incisions than the EXAI group and caused fewer damage to the patient. In fact, we demonstrated that specimen extraction using EXAI was inferior to specimen extraction via EXSI and EVER in terms of short-term postoperative efficacy, postoperative hospital stays, and time to stoma closure.

As treatment became more standardized, the proportion of patients receiving preoperative neoadjuvant radiotherapy increased, and the use of protective ileostomy was more common in patients receiving radiotherapy than in patients not receiving radiotherapy (87.1% vs. 62.5%, P < 0.001) [14]. In our hospital, rectal cancer patients receive radiation therapy usually in combination with single-agent capecitabine to increase radiosensitivity. In addition, multiple studies have shown that patients with obstructive rectal cancer have an increased risk of postoperative anastomotic leakage and are more likely to undergo PLI [15, 16] Therefore, to make our conclusions more convincing, we included patients who received preoperative neoadjuvant chemoradiotherapy and who had intestinal obstruction. Except for the variable tumor distance to lower resection margin, there were no statistically significant difference (P > 0.05) in baseline variables between the groups. The distance of the tumor from the anal verge in the EVER group (4.4 ± 1.1 cm) was the shortest among the three groups. This is because for low or ultra-low rectal cancer patients, the anus valgus technique is used to close and disconnect under direct vision, which not only preserves the anus but also ensures a safe lower incision margin [17].

In the short-term efficacy analysis after primary tumor surgery (the first surgery), patients in the EXAI group had more abdominal auxiliary incisions in the surgical procedure than the EXSI group and EVER group, which increased the surgical trauma and operative time, resulting in more intraoperative blood loss, longer hospital stay, and longer time for early recovery of eating and defecation. For primary surgery, the total postoperative complication rate in the EXAI group was higher than that in the EXSI group (27.9% vs. 10.6%, P = 0.002), and the proportion of complications related to auxiliary incision was 6.7% (7/29) and 0% (0/12), respectively. This indicated that increased auxiliary incision may increase the incidence of postoperative complications and slow down the postoperative recovery speed of the patients [18]. There were also significant differences in complications between EXSI and the EVER group (10.6% vs. 26.1%, P = 0.028), this may be because the anastomotic site of the EVER group is closer to the anus and the number of patients enrolled was too small. However, in our center’s large sample data, 3651 patients with rectal cancer who underwent natural orifice specimen extraction surgery (NOSES) with transanal specimen extraction, the postoperative complication rate was 15.3% [13]. However, Wolthuis et al. conducted a randomized controlled study to compare the early complications after NOSES without auxiliary abdominal incision and traditional laparoscopic surgery and found that the postoperative complication rate of each group was 15% (3/20), but the NOSES group had less postoperative pain and lower analgesic requirements [19]. In the analysis of stoma-related complications, there was no statistically significant difference among the three groups (P = 0.712), indicating that both methods of stoma are safe. Fecal dermatitis was the most common stoma-related complication in all groups (15.4% vs. 20.2% vs. 6.7%). Fecal or urine irritant contact dermatitis was common with ostomies, with more than one-third of colostomy patients and more than two-thirds of ileostomy patients developing peristomal dermatoses [20]. The survival outcome is another focus issue of this study. Especially, during the process of taking the tumor out during EVER, tumor breakage is more likely to occur than in the other two groups. Over the past three decades, with a standard TME technique and selective neoadjuvant chemoradiotherapy in the treatment of rectal cancer, the LRR after radical surgery has fallen to 5–10% [21]. Our study showed that the 3-year LRR of all patients was 4.7%, and there is no statistically different in three groups. Together, the results confirmed that the different specimen extraction techniques do not the risk factor of LRR in low rectal cancer patients.

The current clinical practice of ileostomy closure is usually after about 3–6 months of the primary surgery. Stoma closure performed after 6 months could lead to atrophy of the distal colon muscle and mucosa, resulting in anal dysfunction [22], but optimal timing for stoma closure in the context of adjuvant chemotherapy is controversial. A multicenter study by Herrle et al. showed that the median time to stoma closure was longer in patients who received adjuvant chemotherapy than in those who did not (3.4 vs. 5.6 months, P < 0.001), and late closure (> 6 months postoperative) was significantly associated with impotence and severe fecal incontinence [23]. In this study, patients in the EXAI group had the longest interval to stoma closure (P = 0.018), with an average interval is about 9 months. However, there was no significant difference in the proportion of stage III patients among the three groups (34.6% vs. 35.6% vs. 28.3%, P = 0.159), means that the number of patients receiving adjuvant therapy after surgery is comparable. The reason may be because additional auxiliary incision affects the time to stoma closure, which needs to be confirmed in future large-scale samples. The operation time for loop ileostomy closure (the second surgery) in the EXAI group was significantly longer than that in the other two groups, but there was no difference in the time to early postoperative flatulence/defecation. An interesting finding was that the mean postoperative time to remove the urinary catheter (5.1 days, P = 0.007) and hospital stay (5.8 days, P < 0.001) was shorter in the EXSI group, which may suggest that specimen extraction through the stoma incision was associated with fewer abdominal symptoms and faster postoperative recovery.

Our study has some limitations. First is the lack of functional outcomes such as postoperative anal function and quality of life. Second, although there was no significant difference at baseline among the groups, it was still impossible to control for differences in surgeons’ procedures and patient selection. Third, owing to the inherent limitations of retrospective studies, further large-sized clinical trials may need to be conducted in the future to validate our findings. We believe that our results provide valuable information regarding specimen extraction for low rectal cancer.

## Conclusion

Our single-center results found that in LAR-PLI surgery for low rectal cancer, the short-term outcomes of specimen extraction through the stoma incision or anus were better than that through the auxiliary incision, but the 3-year LRFS was no statistically different.

## Data Availability

The data used to support the findings of this study are available from the corresponding author upon request.
